# Retracted: Successful Treatment of Refractory Wart with a Topical Activated Vitamin D in a Renal Transplant Recipient

**DOI:** 10.1155/2022/9838495

**Published:** 2022-02-23

**Authors:** Case Reports in Transplantation

*Case Reports in Transplantation* has retracted the article titled “Successful Treatment of Refractory Wart with a Topical Activated Vitamin D in a Renal Transplant Recipient” [1], because it contains a duplicated figure with an article published in *Journal of Dermatology* [2]. It was identified that Figure 1 in [1] appears to be identical to Figure 3 in [2], despite differences in the description of the figures.

The authors were contacted for clarification, but they did not respond. The article is therefore retracted from the journal with the agreement of the Editorial Board due to concerns regarding the reliability of the data.
